# Pathogen disgust is associated with interpersonal bias among healthcare professionals

**DOI:** 10.1093/emph/eoad036

**Published:** 2023-10-28

**Authors:** Anastasia Makhanova, W Allen Lambert, Ryan Blanchard, Joe Alcock, Eric C Shattuck, Michael P Wilson

**Affiliations:** Department of Psychological Science, University of Arkansas, 216 Memorial Hall, Fayetteville, AR 72701, USA; Department of Psychological Science, University of Arkansas, 216 Memorial Hall, Fayetteville, AR 72701, USA; Department of Psychological Science, University of Arkansas, 216 Memorial Hall, Fayetteville, AR 72701, USA; Department of Emergency Medicine, University of New Mexico, Albuquerque NM 87131, USA; Department of Anthropology, Florida State University, 60 N Woodward Ave, Tallahassee FL 32304, USA; Institute for Health Disparities Research, College for Health, Community, and Policy, University of Texas at San Antonio, One UTSA Circle, San Antonio, TX 78249, USA; Department of Emergency Medicine, University of Arkansas for Medical Sciences, 4301 W Markham St, Little Rock, AR 72205, USA

**Keywords:** disgust, bias, behavioral immune system, health disparities

## Abstract

**Background and objectives:**

Pathogen avoidance is a fundamental motive that shapes many aspects of human behavior including bias against groups stereotypically linked to disease (e.g. immigrants, outgroup members). This link has only been examined in convenience samples and it is unknown how pathogen avoidance processes operate in populations experiencing prolonged and heightened pathogen threat such as healthcare professionals. We examined whether healthcare professionals demonstrate the same link between pathogen disgust and intergroup bias as has been documented among the general population.

**Methodology:**

Participants (*N* = 317; 210 healthcare professionals) were recruited using snowball sampling to take an online survey. Participants completed the Three Domain Disgust Scale to assess pathogen, sexual and moral disgust. Participants then rated their perceptions of a fictitious immigrant group (‘Krasneeans’) and the degree to which they endorsed group-binding moral values.

**Results:**

Compared to control participants, healthcare professionals reported lower levels of pathogen disgust, but not sexual or moral disgust. However, regardless of profession, higher pathogen disgust was associated with viewing Krasneeans as less likeable and more unclean. Additionally, regardless of profession, higher pathogen disgust was associated with greater endorsement of group-binding moral values, although healthcare professionals reported greater overall endorsement of group-binding moral values than did control participants.

**Conclusions and implications:**

Although healthcare professionals demonstrated lower levels of pathogen disgust, they nevertheless exhibited largely the same relationship between pathogen disgust and interpersonal biases as did control participants. One practical implication of this association is that pathogen avoidance motives may contribute to inequitable patient treatment in healthcare settings.

## 1. INTRODUCTION

The recurrent threat posed by pathogens throughout human evolutionary history has shaped psychological processes to help people avoid potential sources of illness. Although people can detect and respond to cues of actual illness (e.g. [1, 2]), research has shown that people react also to *heuristic* cues that are indirectly related (if at all) to disease (e.g. [3]). Indeed, heightened pathogen avoidance is associated with bias against people who are elderly [4], obese [5], physically atypical [6] or who are perceived as foreign [7]. Although these biases are well-documented in convenience samples, it is unknown how pathogen avoidance processes operate in populations experiencing prolonged and heightened pathogen threats such as healthcare professionals. We examined whether healthcare professionals demonstrate biases related to pathogen avoidance.

Pathogen avoidance influences perceptions of group boundaries and intergroup attitudes. One of the most-documented biases stemming from heightened pathogen avoidance is prejudice against people from racial and ethnic outgroups [7–9]. For example, people with higher trait pathogen avoidance were more likely—compared to those lower in pathogen avoidance—to rate an immigrant group from a fictitious Central African country (‘Krasnee’) as less likeable and trustworthy, as well as to say that Krasneeans should not be allowed to immigrate to their country [7]. More generally, trait pathogen avoidance is associated with greater sensitivity to intergroup boundaries, including novel boundaries between lab-created groups [4]. Additionally, people with higher trait pathogen avoidance tend to espouse moral values that function to bind the group together by maintaining ingroup loyalty, hierarchy and sanctity [10–12].

Beyond individual differences in pathogen avoidance, recent research has suggested that life events can affect pathogen avoidance. Decreased pathogen disgust has been observed in military cadets after exposure to the harsh conditions of a 10-day military camp, suggesting attenuated pathogen avoidance in those circumstances [13]. However, environmental conditions can also elevate pathogen disgust, such as in Polish women during the COVID-19 pandemic [14]. Momentary increases in pathogen avoidance may also occur when people see images of others who are ill or read articles about impending illness threats [15]. Little is known, however, about how prolonged exposure to environmental stimuli that signal the presence of pathogens affects both pathogen avoidance and resulting biases.

Healthcare professionals are particularly relevant for examining pathogen avoidance effects because they are inundated with pathogen threat cues and, perhaps in consequence, report lower pathogen disgust than the general population [16, 17]. This lower disgust could be due to desensitization of pathogen disgust and/or to self-selection of low-disgust individuals into medical professions. In support of the desensitization hypothesis Rozin [16] demonstrated that first-year medical students become less likely to rate cadavers as a disgust-eliciting stimulus during their cadaver labs [16]. Conversely, undergraduate students with lower disgust sensitivity report more interest in careers that involve direct patient care or more contact with disgusting stimuli (pre-medical and pre-nursing) compared to careers that involve little to no patient care and little contact with disgusting stimuli (pre-pharmacy [18]).

Do lower levels of disgust among healthcare professionals have implications for the relationship between pathogen avoidance and social biases? On one hand, lower disgust may mean lower pathogen avoidance motivation, which may translate to lower social bias or a weak association between pathogen avoidance and bias. On the other hand, healthcare professionals are not immune to the effects of the pathogen avoidance system. A recent survey showed that healthcare workers in a COVID-19 hospital who had elevated levels of pathogen disgust also had a greater fear of infection [19]. Thus, examining whether pathogen avoidance is associated with social bias in healthcare professionals may have implications for healthcare equity, because pathogen avoidance may be one contributing factor to treatment disparities for patients from racial and ethnic minoritized groups.

### 1.1. Current research

We examined whether the association between pathogen avoidance and social bias emerged among healthcare professionals, who encounter cues of pathogen threat much more frequently than the general population. Both healthcare professionals and non-medical control participants completed an online survey assessing trait pathogen avoidance via the Three Domain Disgust scale (i.e. pathogen, moral and sexual disgust [20]) and two measures of intergroup bias previously linked with pathogen avoidance: perceptions of a fictitious immigrant group [7] and endorsement of group-binding moral foundations ([10–12, 21]). We hypothesized that we would replicate the link between pathogen disgust and social bias in the entire sample and explored whether this link would be moderated by work in medicine.

We additionally examined whether healthcare professionals differed from control participants in terms of disgust sensitivity and hypothesized that differences would be domain specific. That is, we predicted that healthcare professionals would report lower pathogen disgust than control participants; we did not predict differences for moral or sexual disgust. Furthermore, we predicted that, among healthcare professionals, both time in career and frequency of contact with contagious patients would be negatively associated with pathogen disgust.

## 2. METHODOLOGY

### 2.1. Participants

Participants (*N* = 348) were recruited online using snowball sampling. Three of the authors first contacted healthcare professionals (broadly defined, but primarily nurses and physicians) in their extended social and professional networks. Participants working in healthcare were asked to share study information with (i) people they knew who also worked a medical field and (ii) someone they knew who was similar to themselves in terms of demographics (age, race and gender) but did not work in a medical field. All participants received a $10 Amazon gift card for participation. We excluded 29 participants because they failed attention checks: 17 participants failed an attention check embedded within the Three Domain Disgust scale, 5 participants failed a second attention check embedded within the Moral Foundations Questionnaire and 7 participants failed a third attention check related to a measure described in the Supplemental Materials).

Participants answered a self-report question (yes/no) regarding current employment in a medical field. However, when we examined the free-response answers, it became evident that some people did not answer these questions in the same way that we were conceptualizing our groups. For example, several medical and nursing students answered no but some clinical psychologists answered yes. Thus, rather than using the self-selected variable, we categorized people using their free-response answers based on whether they were currently undergoing or have completed professional training in a medical field (nursing school, medical school, speciality certification, etc.). Two authors completed the first round of categorization and the first author subsequently served as a tie breaker for discrepancies. Of the 319 participants who passed attention checks, we categorized 210 as healthcare professionals, 107 as control participants and 2 participants were excluded because they did not provide answers to the free response questions about their current profession. On average, healthcare professionals worked in their field for 9 years (*M* = 9.78, SD = 9.65, range: 0–48). Healthcare professionals additionally reported how often they encounter people who are contagious on a scale of 1 (*Never*) to 7 (*Multiple times per day*). Consistent with the fact that data were collected during the COVID-19 pandemic—between 13 November 2020 and 16 March 2021—most healthcare professionals reported encountering contagious people at least several times per week (*M* = 5.77, SD = 1.63).

Participants in the final sample (*n* = 317) were on average 33 years old (*M* = 33.73, SD = 11.78, range: 18–74). Most participants identified their gender as female (*n* = 258), 57 as male, and 1 did not wish to report. Notably, gender was not equally distributed across professions, *χ*^2^(2) = 15.82, *P* < 0.001; there was a larger proportion of women among healthcare professionals (87.6%) than in the control group (69.2%). Most participants self-reported their race as White/Caucasian (260, 82%), 25 participants self-reported their race as Asian, 12 as Black/African American, 2 as Native American, 10 as multiracial or other and 5 did not wish to report. Eighteen participants identified their ethnicity as Hispanic. On a scale of 1 (*Extremely liberal*) to 10 (*Extremely conservative*), participants were on average around the midpoint for political orientation (*M* = 5.43, SD = 2.64); on a scale of 1 (*Not religious at all*) to 10 (*Extremely religious*), participants were on average around the midpoint for religiosity (*M* = 5.96, SD = 2.97).

### 2.2. Procedure and materials

After providing informed consent, participants completed the main dependent measures presented in random order.

*Disgust sensitivity.* Participants completed the Three Domain Disgust Scale [20], comprised of 21 items that measure individuals’ disgust sensitivity within the three domains of disgust: pathogen disgust (e.g. ‘Stepping on dog poop’; α = .77), sexual disgust (e.g. ‘Hearing two strangers have sex’; α = .82) and moral disgust (e.g. ‘Stealing from a neighbor’; α = .81). Participants rated the extent to which they found each scenario disgusting on a scale from 1 (*Not disgusting at all*) to 7 (*Extremely disgusting*).

*Outgroup bias*. To examine the associations between pathogen disgust and perceptions of a fictitious immigrant group, we asked participants to read a short passage about a Central African country named Krasnee, adapted from the description provided in past research [7]; Study 2). Notably, the passage indicated that 100 Krasneeans have completed immigration paperwork to move to the USA. This number was chosen based on vignettes used in prior research [22]. The full text of the vignette can be found in the Supplemental Materials. After reading the passage, participants were first asked to rate their perceptions of Krasneeans on several characteristics (likeable, hostile, trustworthy, open-minded, ignorant, poor and unclean) on a scale from 1 (*Not at all*) to 7 (*Very much*). Using this measure Faulkner *et al.* [7] demonstrated that people with higher trait levels of pathogen avoidance rated Krasneeans as less likeable and less trustworthy than those with lower trait levels of pathogen avoidance. (We added ‘unclean’ although it was not one of the adjectives used by Faulkner *et al.* [7].) We hypothesized that we would replicate these findings and examined whether healthcare professionals would show similar effects.

Next, we assessed participants’ attitudes toward Krasneean immigration. Participants indicated how many Krasneeans should be allowed to immigrate to the United States on a scale of 0 (*None*) to 4 (*All*). (The wording of this item was changed to increase comprehension. The original item was negatively worded—‘None of the Krasneeans applying for citizenship should be allowed to immigrate to Canada’—which typically is a more difficult question format for participants to answer because of the double-negative.). Participants also rated their agreement with the statement ‘The United States’ immigration policies are too strict’ on a scale of 1 (*Strongly disagree*) to 7 (*Strongly agree*) Faulkner *et al.* [7] demonstrated that people with higher trait levels of pathogen avoidance were more likely to say that none of the Krasneeans should be allowed to immigrate (to Canada, in that survey) compared to those with lower trait levels of pathogen avoidance; no associations were documented for general anti-immigration attitudes. We hypothesized that we would replicate these findings. Additionally, participants answered yes/no to the question ‘If the decision were up to you, would you let Krasneeans immigrate to the United States?’ Ji *et al.* [22] demonstrated that higher trait pathogen avoidance was associated with deciding not to let a group of 100 immigrate to the USA, even if they did not receive information about the population health background of that immigrant group. We hypothesized that we would replicate these findings and examined whether healthcare professionals would show similar effects.

Finally, we created exploratory measures to assess whether participants’ comfort with Krasneean immigration could be increased with additional screenings or assurances. Full measure descriptions and results can be found in the Supplemental Materials.

*Endorsement of moral values*. To assess participants’ endorsement of different domains of morality, participants completed the Moral Foundations Questionnaire (MFQ [23]). The MFQ consists of 30 items with 6 items per each of the 5 domains: care (e.g. ‘One of the worst things a person could do is hurt a defenseless animal’; *α* = .58), fairness (e.g. ‘Justice is the most important requirement for a society’; *α* = .63), ingroup loyalty (‘It is more important to be a team player than to express oneself’; *α* = .73), authority (‘Respect for authority is something all children need to learn’; *α* = .75) and sanctity (e.g. ‘People should not do things that are disgusting, even if no one is harmed’; *α* = .79). Participants answered on a scale of 1–7 with higher values indicating greater endorsement of each moral domain. Following past research, we focused on the two superordinate factors of individualizing moral values (care and fairness; *α* = .76) and group-binding moral values (ingroup loyalty, obedience to authority, and sanctity; *α* = .89). Past research has linked pathogen disgust to greater endorsement of group-binding moral values ([10–12]; van & Park, 2009).

Participants additionally completed a measure of social categorization bias for elderly or young targets that is beyond the scope of the current manuscript. Full measure descriptions and results can be found in the Supplemental Materials.

## 3. RESULTS

### 3.1. Descriptive results

See Table 1 for comparisons of age, political orientation, and religiosity between healthcare professionals and control participants. There was a trend for healthcare professionals to report more conservative political orientation and greater religiosity relative to control participants. Notably, the group differences in political orientation appeared to be mediated by sexual disgust (but not pathogen or moral disgust) whereas the group differences in religiosity appeared to be mediated by both sexual and pathogen disgust (but not moral disgust). The results of these models are reported in the Supplemental Materials.

**Table 1.** Comparison of demographic variables between participant groups

Response	Control Participants	Healthcare Professionals	*t*	df	*P*	*d*
	*M*(SD)	*M*(SD)				
Age	34.03(12.67)	33.58(11.32)	0.32	314	0.746	0.04
Political orientation	5.08(2.64)	5.61(2.63)	−1.68	314	0.096	0.20
Religion	5.53(3.28)	6.18(2.78)	−1.74	185.29	0.084	0.21

Correlations between demographic variables and disgust sensitivity are reported in Table 2 (see Supplemental Materials for correlations between variables within each group). Notably, unlike prior research [20, 24, 25], pathogen disgust was not associated with religiosity, political orientation, or gender either across the entire sample or within groups. The absence of a correlation between pathogen disgust and these demographic variables may be because these data were collected during the COVID-19 pandemic. The pandemic may have influenced these results either because of high levels of pathogen threat experienced by all people or because of the politicization of the pandemic response in the USA. Consistent with past research, women reported greater sexual disgust [20, 26]; sexual disgust was also significantly correlated with political orientation and religiosity [20, 27].

**Table 2.** Correlations between demographic variables and disgust sensitivity

Variable	1	2	3	4	5	6
1. Age						
2. Gender (0 = male, 1 = female)	−.07					
3. Political orientation	.14*	.02				
4. Religiosity	.08	.05	.52***			
5. Pathogen disgust	.03	.03	.04	−.02		
6. Sexual disgust	.06	.30***	.36***	.46***	.31***	
7. Moral disgust	.33***	.02	.17**	.19**	.19**	.31***

Higher values for political orientation represent more conservative orientations. Higher values for religiosity represent stronger religiosity. *** *P* < 0.001, ** *P* < 0.01, * *P* < 0.05.

### 3.2. Descriptive results: differences in disgust sensitivity and bias

We conducted three independent samples *t*-tests to examine differences in pathogen, sexual and moral disgust sensitivity between healthcare professionals and participants in the control group working in other professions (see Table 3). Consistent with predictions, healthcare professionals reported lower pathogen disgust than the control group. Healthcare professionals reported *higher* sexual disgust and a trend for higher moral disgust than the control group. Given that previous research has indicated that women demonstrate higher sexual disgust than men [28], this finding may be in part due to the greater proportion of women in the healthcare professional group compared to the control group. Consistently, in ancillary analyses that controlled for gender (male = 0, female = 1)—as well as age, political orientation and religiosity—the group differences were no longer significant (see Supplemental Materials). Thus, it appears that the only robust difference in disgust sensitivity between the two groups is within the pathogen domain.

**Table 3.** Differences in disgust sensitivity and bias between participant groups

Variable	Control Participants	Healthcare Professionals	*t*	*df*	*p*	*d*
	*M*(SD)	*M*(SD)				
Disgust domain						
Moral disgust	5.16(1.14)	5.41(0.90)	−1.96	175.80	0.052	−0.24
Sexual disgust	**3.88(1.44)**	**4.28(1.25)**	−**2.56**	**315**	**0.011**	−**0.30**
Pathogen disgust	**4.68(1.06)**	**4.32(1.07)**	**2.85**	**315**	**0.005**	**0.34**
Evaluation of Krasneeans						
Likeable	4.33(0.88)	4.43(1.05)	−0.82	243.43	0.440	−0.09
Trustworthy	4.17(0.89)	4.14(1.02)	0.22	312	0.927	0.03
Open-Minded	4.26(1.28)	4.19(1.37)	0.41	311	0.686	0.05
Ignorant	3.53(1.17)	3.43(1.27)	0.68	312	0.498	0.08
Poor	4.52(1.49)	4.49(1.41)	0.17	312	0.868	0.02
Unclean	3.69(1.31)	3.54(1.43)	0.88	312	0.382	0.10
Hostile	3.51(1.12)	3.56(1.28)	−0.33	312	0.742	−0.04
Moral foundation endorsement						
Individualizing	5.49(0.74)	5.39(0.78)	1.16	315	0.246	0.14
Harm/Care	5.60(0.75)	5.55(0.87)	0.55	315	0.584	0.07
Fairness	5.39(0.86)	5.23(0.84)	1.56	315	0.119	0.18
Binding	**4.11(1.16)**	**4.38(1.00)**	−**2.04**	**186.90**	0**.043**	−**0.25**
Ingroup Loyalty	**3.88(1.19)**	**4.19(1.10)**	−**2.32**	**315**	0**.021**	−**0.27**
Authority	4.36(1.23)	4.57(1.04)	−1.47	184.66	0.145	−0.18
Purity/Sanctity	4.10(1.46)	4.39(1.27)	−1.75	189.37	0.081	−0.21

Effects that were significant at α = .05 appear in bold.

We next examined zero-order correlations to test the predictions that frequency of contact with contagious patients and time in career would negatively correlate with pathogen disgust. Consistently, contagious patient contact frequency was negatively associated with pathogen disgust, *r*(197) = −0.18, *P* = 0.011, but not with sexual disgust, *r*(197) = −0.10, *P* = 0.173, or moral disgust, *r*(197) = 0.04, *P* = 0.585. The association with pathogen disgust was robust even when controlling for covariates (age, gender, political orientation and religiosity in a multiple linear regression model, *b* = −0.12, SE = 0.05, *t*(189) = −2.42, *P* = 0.016, 95% CI [−0.21, −0.02], semi-partial *r* = −0.17. Although time in career was not associated with healthcare professionals’ pathogen disgust, *r*(197) = −0.06, *P* = 0.413, or sexual disgust, *r*(197) = 0.02, *P* = 0.834, time in career was positively associated with moral disgust, *r*(197) = 0.24, *P* = 0.001. However, in regression analyses that included covariates, it appeared that this association may have been driven by participant age, *b* = 0.02, SE = 0.01, *t*(189) = 1.96, *P* = 0.051, 95% CI [−0.0001, 0.044], semi-partial *r* = 0.13, and not time in medicine specifically, *b* = −0.003, SE = 0.01, *t*(189) = −0.24, *P* = 0.808, 95% CI [−0.03, 0.02], semi-partial *r* = −0.02.

We also conducted independent samples *t*-tests to examine differences in key social bias outcomes. Group differences emerged only for endorsement of binding moral foundations generally and ingroup loyalty moral values specifically; healthcare workers demonstrated greater endorsement of these moral values than control participants.

### 3.3. Association between pathogen disgust and perceptions of Krasneeans

We tested whether we replicated the previously documented association between pathogen avoidance and perceptions of Krasneeans in our overall sample and whether this association was affected by medical work by modeling the relationship of participant group (control = −0.5, healthcare professionals = 0.5), pathogen disgust (*z*-scored), and the group by pathogen disgust interaction on participants’ perceptions of Krasneeans (see Table 4). Overall, participants with higher (versus lower) pathogen disgust rated Krasneeans as less likeable and more unclean, with a nonsignificant trend for more ignorant. Perceptions of hostility were moderated by group: although control participants with higher (versus lower) pathogen disgust perceived Krasneeans as more hostile, among healthcare professionals there was no association between pathogen disgust and perceptions of hostility. Pathogen disgust was not associated with perceptions of Krasneeans as trustworthy, open-minded or poor. The pattern of associations was largely robust to the inclusion of covariates (see Supplemental Materials).

**Table 4.** Associations between pathogen disgust and perceptions of Krasneeans

Characteristic	*b* (SE)	*t*	*p*	95% CI	*r*
Likeable	−**0.14 (0.06)**	−**2.24**	**.026**	**[**−**0.24,** −**0.02]**	−**0.13**
Trustworthy	−0.03 (0.06)	−0.48	.629	[−0.15, 0.09]	−0.03
Open-minded	0.03 (0.08)	0.38	.706	[−0.13, 0.19]	0.02
Ignorant	0.15 (0.08)	1.94	.053	[−0.002, 0.29]	0.11
Poor	0.04 (0.09)	0.48	.632	[−0.13, 0.21]	0.03
Unclean	**0.27 (0.08)**	**3.25**	**.001**	**[0.11, 0.44]**	**0.18**
Hostile (PD × Group)	−**0.31 (0.15)**	−**2.12**	**.035**	**[**−**0.60,** −**0.02]**	−**0.12**
Healthcare Professionals	0.12 (0.09)	1.36	.175	[−0.05, −0.28]	0.08
Control Participants	**0.43 (0.12)**	**3.56**	** < 0.001**	**[0.19, 0.66]**	**0.20**

*df* = 310, *r* is the semi-partial *r*, except for perceptions of Krasneeans as hostile, all other interactions between pathogen disgust and group were not significant (*P*s ≥ 0.599).

Effects that were significant at α = .05 appear in bold.

Next, we examined whether pathogen disgust was associated with attitudes toward Krasneean immigration and whether this association was moderated by the participant group. For the amount of Krasneeans that should be allowed to immigrate (answer options ranging from none to all), there was a trend for a negative association with pathogen disgust, *b* = −0.13, SE = 0.08, *t*(311) = −1.66, *P* = 0.098, 95% CI [−0.27, 0.02], semi-partial *r* = −0.09. There were no group differences, *b* = −0.17, SE = 0.15, *t*(311) = −1.14, *P* = 0.257, 95% CI [−0.47, 0.13], semi-partial *r* = −0.06, and the association between pathogen disgust and attitudes toward Krasneean immigration was not moderated by group, *b* = −0.01, SE = 0.15, *t*(311) = −0.05, *P* = 0.960, 95% CI [−0.30, 0.29], semi-partial *r* = −0.003. The association with pathogen disgust and attitudes toward Krasneean immigration, however, became non-significant in analyses that included the covariates, *b* = −0.09, SE = 0.07, *t*(303) = −1.26, *P* = 0.208, 95% CI [−0.23, 0.05], semi-partial *r* = −0.07.

Only 37 participants said that they would not allow the Krasneeans to immigrate; in a logistic regression, the decision was not associated with pathogen disgust, group, or the pathogen disgust by group interaction (all *P*’s ≥ 0.222). For general perceptions of immigration policies, there was no association with pathogen disgust, *b* = −0.08, SE = 0.12, *t*(311) = −0.64, *P* = 0.520, 95% CI [−0.32, 0.16], semi-partial *r* = −0.04. However, healthcare professionals were less likely than control participants to say that the US immigration policies are too strict, *b* = −0.58, SE = 0.24, *t*(311) = −2.41, *P* = 0.017, 95% CI [−1.05, −0.11], semi-partial *r* = −0.14, but this difference was no longer apparent after controlling for covariates, *b* = −0.25, SE = 0.17, *t*(303) = −1.46, *P* = 0.145, 95% CI [−0.59, 0.09], semi-partial *r* = −0.06.

In ancillary analyses (see Supplemental Materials), we found that pathogen disgust was positively associated with thinking that it would be important for Krasneeans to undergo additional health, financial and niceness screenings, but not criminal screening. We additionally found that participants with higher pathogen disgust, compared to those with lower pathogen disgust, would have felt more comfortable with Krasneean immigration to the extent they received assurances that (a) they would not have any contact with the Krasneeans, (b) knew that the immigrating Krasneeans would adopt American norms and culture or (c) knew that the immigrating Krasneeans would come from an area without any viruses.

### 3.5. Association between pathogen disgust and moral foundations

Finally, we tested whether we replicated the previously documented association between pathogen avoidance and endorsement of binding moral foundations in our overall sample and whether this association was affected by medical work by modeling the relationship of participant group, pathogen disgust and the group by pathogen disgust interaction on the endorsement of binding moral foundations, controlling for endorsement of individualizing moral foundations. Supporting our hypotheses, pathogen disgust was positively associated with endorsement of binding moral foundations, *b* = 0.28, SE = 0.06, *t*(312) = 4.49, *P* < 0.001, 95% CI [0.16, 0.41], semi-partial *r* = 0.24. Although we found that healthcare professionals were more likely to endorse binding moral foundations relative to control participants, *b* = 0.37, SE = 0.12, *t*(312) = 3.00, *P* = 0.003, 95% CI [0.13, 0.61], semi-partial *r* = 0.16., medical work did not moderate the association between pathogen disgust and endorsement of binding moral foundations, *b* = −0.06, SE = 0.12, *t*(312) = −0.49, *P* = 0.622, 95% CI [−0.31, 0.18], semi-partial *r* = −0.03. Both main effects of work in medicine and pathogen disgust were robust to the inclusion of covariates. Descriptive statistics for between-group differences in moral foundation endorsement—each foundation separately and the superordinate factors—are reported in the Supplemental Materials.

We ran a similar model to examine associations with endorsement of individualizing moral foundations. Pathogen disgust was positively associated with endorsement of individualizing moral foundations as well, *b* = 0.15, SE = 0.05, *t*(312) = 3.06, *P* = 0.002, 95% CI [0.05, 0.24], semi-partial *r* = 0.17. However, there were no group differences, *b* = −0.06, SE = 0.09, *t*(312) = 0.70, *P* = 0.487, 95% CI [−0.24, 0.12], semi-partial *r* = −0.04, and the association between pathogen disgust and endorsement of individualizing moral foundations was not moderated by group, *b* = −0.01, SE = 0.09, *t*(312) = −0.11, *P* = 0.916, 95% CI [−0.19, 0.17], semi-partial *r* = −0.01. Notably, the association between pathogen disgust and endorsement of individualizing moral foundations was no longer significant after the inclusion of the covariates, *b* = 0.07, SE = 0.04, *t*(304) = −1.58, *P* = 0.116, 95% CI [−0.02, 0.15], semi-partial *r* = 0.08.

### 3.6. Sexual disgust and bias

Although the primary focus of the present research was pathogen disgust, research suggests that sexual disgust is also associated with social bias [29–31]. We conducted ancillary exploratory analyses to examine whether any unique effects of sexual disgust emerged for perceptions of Krasneeans and endorsement of moral foundations. Full results are reported in the Supplemental Materials. We found that among healthcare workers, but not control participants, sexual disgust was negatively associated with perceiving the Krasneeans as likeable. No other perceptions of Krasneeans were associated with sexual disgust. The association between sexual disgust and endorsement of binding moral foundations was also moderated by group. In this case, however, it was among control participants that sexual disgust was associated with greater endorsement of binding moral foundations; the association was not significant among healthcare professionals. These findings suggest that something about healthcare professionals’ experiences or traits may affect the way sexual disgust influences their social perceptions.

## 4. DISCUSSION

We examined whether healthcare professionals differed from people working in other professions in pathogen disgust and in the association between pathogen disgust and social biases. Consistent with our hypotheses and prior research [16–18], healthcare professionals reported lower pathogen disgust than control participants. However, the two groups did not differ in two other domains of disgust: sexual and moral disgust. These findings are consistent with research suggesting that one’s circumstances can reduce sensitivity in specific relevant domains of disgust (e.g. [13, 14, 16]).

In this study, healthcare professionals were largely similar to non-healthcare workers in showing an association between social bias and pathogen disgust. When evaluating a fictional immigrant group of Krasneeans, regardless of group, participants with higher pathogen disgust rated the group as less likeable and more unclean. These findings are generally consistent with the original findings of Faulkner *et al.* [7] who found that trait pathogen avoidance (operationalized as perceived vulnerability to disease) was negatively associated with ratings of Krasneeans as likeable and open-minded. In both studies, pathogen avoidance was associated with more negative perceptions of Krasneeans, even though the specific traits were different. Notably, ratings of pathogen disgust were positively associated with perceptions of Krasneeans as hostile among control participants, but there was no such association between pathogen disgust and hostility among healthcare professionals.

Healthcare professionals were no different from control participants in the second measure of intergroup bias—endorsement of group-binding moral values (i.e. ingroup loyalty, obedience to authority and purity [23]. In both groups, pathogen disgust was positively associated with endorsement of group-binding moral values, but not related to endorsement of individualizing moral values (i.e. harm and fairness). This is consistent with past research linking endorsement of group-binding moral values with individual-level variability in trait pathogen avoidance [10–12] and country-level variability pathogen threat [32].

Taken together, our findings suggest that healthcare professionals demonstrate the same relationship between pathogen disgust and bias as in people working in other professions, even though healthcare professionals have lower pathogen disgust overall. Although we did not assess the downstream consequences of this bias, pathogen disgust may contribute to the well-documented treatment disparities experienced by immigrants who seek healthcare in the USA. For example, undocumented Hispanic immigrants take longer to receive HIV care than other populations [33], and third-generation or greater Mexican Americans were four times more likely to be prescribed opioids to treat their chronic pain compared to first-generation Mexican Americans [34]. It is worth noting that pathogen avoidance can also contribute to bias against patients from other groups heuristically associated with disease such as people who are elderly [35, 15] or overweight [5, 15, 36, 37]. Future research should directly examine how pathogen avoidance may contribute to biased patient care decisions and health disparities.

### 4.1. Limitations and future directions

The cross-sectional design of this study does not permit causal inferences about group differences in pathogen disgust sensitivity. One possibility is that frequent exposure to pathogen threat leads to desensitization, such as the findings of [16] on the desensitization of first-year medical students while working with cadavers. This desensitization was notably specific to the particular facet of disgust relating to dead bodies. However, as the present research only examined general pathogen disgust, future research might benefit from a more fine-grained approach to test whether healthcare professionals become generally desensitized or particularly desensitized to the pathogen cues that they encounter in their day-to-day practice.

A second possible explanation that cannot be ruled out by these data is that people with lower disgust sensitivity may be more likely to self-select into careers that require contact with disgust-eliciting stimuli [18]. Thus, the observed differences may have existed prior to participants deciding on their career path rather than being the result of work within their careers. Future research would benefit from a longitudinal design that would be able to adjudicate between self-selection and desensitization.

The generalizability of the present findings is limited by the use of snowball sampling. This nonprobability sampling approach can lead to biased sample selection because people may share the survey with others who are more similar to them and avoid sharing the survey with others who are less similar to them. Consequently, our sample may be more homogenous than the population of interest. Moreover, snowball sampling can also introduce dependencies in the data or problems with restricted range. To enhance the external validity of the link between working in healthcare and disgust, future research should use more rigorous probability sampling techniques.

Although we found that pathogen disgust was associated with more negative perceptions of the Krasneeans, this measure has several limitations. First, our results are only somewhat consistent with the original findings Faulkner *et al.* [7] found that participants’ scores on the perceived vulnerability to disease questionnaire, another commonly used measure of trait pathogen avoidance, was negatively associated with perceiving Krasneeans as likeable and trustworthy. In our study, we conceptually replicated the negative association between pathogen avoidance and perceptions of Krasneeans as likeable, but we did not observe a negative association between pathogen avoidance and perceptions of Krasneeans as trustworthy. We did, however, additionally observe associations between pathogen disgust and perceptions of Krasneeans being unclean, which was a new perception added for the present study. Notably, our effect sizes are somewhat smaller than those reported by [7], which may be partially due to inflated effect sizes in the original study due to its smaller sample size or sociocultural differences between the two samples.

Second, in the present study, Krasneeans were only described as being from Central Africa. The link between pathogen avoidance and social bias is modulated by factors such as subjective familiarity with the group’s culture [7], information about the level of pathogen threat in the group’s former ecology [22], and situational increases in the association between pathogen threat and particular groups (Makhanova, 2022). It would be important to examine how such pathogen avoidance is associated with healthcare professionals’ bias against other groups and how bias may be influenced by patient demographics in their clinic. Furthermore, some pathogen threats are uniquely linked to specific minoritized groups. It would be important to examine whether this linkage contributes to disgust-related biases for these groups (e.g. gay men and HIV), perhaps in specific contexts when the threat is activated (e.g. contact with blood).

Finally, because this measure did not have a control group, we cannot distinguish between the hypothesis that pathogen disgust is associated with outgroup-specific prejudice or the alternative hypothesis that pathogen disgust is associated with general avoidance. Although the link between pathogen avoidance and anti-immigrant prejudice is robust (e.g. [7–9, 38], the theoretical explanation for this link is debated (e.g. [22, 39, 40]). Moreover, pathogen disgust does reduce people’s general desire to affiliate [41, 42].

## 5. CONCLUSION

We investigated how working in healthcare affected people’s disgust sensitivity and the association between pathogen disgust sensitivity and social bias. Healthcare workers tended to report lower levels of pathogen disgust, especially if they reported frequent contact with contagious patients. Nevertheless, the associations between disgust sensitivity and (a) perceptions of a fictional immigrant group and (b) endorsement of binding moral foundations were largely similar between control participants and healthcare professionals. These findings suggest that pathogen avoidance may contribute to disparities in treatment received by patients from minoritized groups.

## Supplementary Material

eoad036_suppl_Supplementary_Material

## Data Availability

Data underlying this article are available on OSF (https://osf.io/8gjmc/). Because the first three authors recruited participants through their personal networks, participant answers to open-ended career fields are not available publicly but will be shared on reasonable request to the corresponding author.
