# Supplementary material for: Pathogen disgust is associated with interpersonal bias among healthcare professionals
Source: Evol Med Public Health. 2023 Oct 28;11(1):438–47. doi: 10.1093/emph/eoad036 (PMC10667654; doi:10.1093/emph/eoad036)
Supplement: eoad036_suppl_Supplementary_Material [file eoad036_suppl_supplementary_material.docx]

**Pathogen Disgust is Associated with Interpersonal Bias Among Healthcare Professionals**

Supplemental Materials

**Table of Contents**

1. [Correlations between demographic variables and disgust sensitivity within each group](#_Correlations_between_demographic)

2. [Differences in disgust controlling for covariates](#_Differences_in_disgust)

3. [Vignette about Krasneean immigration](#_Vignette_about_Krasneean)

4. [Evaluation of Krasneeans controlling for covariates](#_Evaluation_of_Krasneeans)

5. [Exploratory measures and results for additional screenings and assurances
that may increase comfort with Krasneean immigration](#_Exploratory_measures_and)

6. [Exploratory analyses examining associations between sexual disgust and Krasneean immigration](#_Exploratory_analyses_examining_1)

7. [Exploratory analyses examining associations between sexual disgust and endorsement of moral foundations](#_Exploratory_analyses_examining_2)

8. [Mediation models to examine whether disgust sensitivity mediates the group differences in religiosity and political orientation](#_Mediation_models_to)

9. [Assessment of categorization bias for elderly targets](#_Assessment_of_categorization_1)

# **Correlations between demographic variables and disgust sensitivity within each group**

Table A1

*Correlations between demographic variables and disgust sensitivity* ***AMONG CONTROL****.*

| Variable | 1 | 2 | 3 | 4 | 5 | 6 |
| --- | --- | --- | --- | --- | --- | --- |
| 1. Age |  |  |  |  |  |  |
| 2. Gender (0=male, 1=female) | .16 |  |  |  |  |  |
| 3. Political Orientation | .14 | -.03 |  |  |  |  |
| 4. Religiosity | .05 | -.04 | .60*** |  |  |  |
| 5. Pathogen Disgust | .14 | .04 | .11 | -.10 |  |  |
| 6. Sexual Disgust | .16 | .18 | .40*** | .43*** | .29** |  |
| 7. Moral Disgust | .38*** | .06 | .17 | .18 | .28** | .42*** |

*Note.* Higher values for political orientation represent more conservative orientations. Higher values for religiosity represent stronger religiosity. *** *p* < .001, ** *p* < .01, * *p* < .05

Table A2

*Correlations between demographic variables and disgust sensitivity* ***AMONG HEALTHCARE****.*

| Variable | 1 | 2 | 3 | 4 | 5 | 6 |
| --- | --- | --- | --- | --- | --- | --- |
| 1. Age |  |  |  |  |  |  |
| 2. Gender (0=male, 1=female) | -.20** |  |  |  |  |  |
| 3. Political Orientation | .15* | .08 |  |  |  |  |
| 4. Religiosity | .10 | .05 | -.47*** |  |  |  |
| 5. Pathogen Disgust | -.03 | .07 | .02 | .05 |  |  |
| 6. Sexual Disgust | -.02 | .31*** | .33*** | .46*** | .37*** |  |
| 7. Moral Disgust | .30*** | -.09 | .15* | .19** | .18** | .20** |

*Note.* Higher values for political orientation represent more conservative orientations. Higher values for religiosity represent stronger religiosity. ***, *p* < .001, ** *p* < .01, * *p* < .05

# **Differences in disgust controlling for covariates**

In ancillary analyses that controlled for age, gender (male = 0, female = 1), political orientation, and religiosity, healthcare professionals demonstrated significantly lower pathogen disgust compared to control participants, *b* = -0.39, *SE* = 0.13, *t*(307) = -2.98, *p* = .003, 95% CI [-0.65, -0.13], semi-partial *r* = -.17. In these analyses, participant groups were not different in terms of sexual disgust, *b* = 0.12, *SE* = 0.14, *t*(307) = 0.84, *p* = .401, 95% CI [-0.16, 0.39], semi-partial *r* = .04, or moral disgust, *b* = 0.22, *SE* = 0.11, *t*(307) = 1.94, *p* = .054, 95% CI [-0.004, 0.446], semi-partial *r* = .12.

# **Vignette about Krasneean Immigration**

There is a country in Central Africa that has been experiencing a great deal of change in recent years. For the purposes of this study, we will refer to this country as Krasnee.

Krasnee was once filled with tribal regions, but now the country is having trouble unifying their citizens. The citizens of Krasnee are experiencing hard times and the situation does not look like it will be getting better any time soon. Many people from this country are interested in leaving Krasnee and immigrating to another part of the world. Indeed, 100 Krasneeans have completed immigration paperwork to move to the United States.

# **Evaluation of Krasneeans controlling for covariates**

Table B

*Associations between pathogen disgust and perceptions of Krasneeans controlling for age, gender, religiosity, and political orientation.*

| Characteristic | *b* (*SE*) | *t* | *p* | 95% CI | *r* |
| --- | --- | --- | --- | --- | --- |
| Likeable | -0.12 (0.06) | -1.91 | .058 | [-0.24, 0.004] | -.11 |
| Trustworthy | -0.01 (0.06) | -0.21 | .836 | [-0.13, 0.10] | -.01 |
| Open-minded | 0.04 (0.08) | 0.50 | .614 | [-0.12, 0.20] | .03 |
| **Ignorant** | **0.14 (0.07)** | **2.08** | **.039** | **[0.01, 0.30]** | **.12** |
| Poor | 0.06 (0.09) | 0.63 | .527 | [-0.12, 0.23] | .04 |
| **Unclean** | **0.28 (0.08)** | **3.32** | **.001** | **[0.11, 0.44]** | **.19** |
| **Hostile (PD x Group)** | **-0.30 (0.15)** | **-2.01** | **.045** | **[-0.59, -0.01]** | **-.11** |
| Healthcare professionals | 0.10 (0.08) | 1.24 | .216 | [-0.06, 0.27] | .07 |
| **Control Participants** | **0.40 (0.12)** | **3.32** | **.001** | **[0.16, 0.64]** | **.18** |

*Note.* *df* = 302, *r* is the semi-partial *r*, except for perceptions of Krasneeans as hostile, all other interactions between pathogen disgust and group were not significant (*p*s ≥ .674).

# **Exploratory measures and results for additional screenings and assurances that may increase comfort with Krasneean immigration**

For screenings, participants were asked to imagine a scenario that Krasneeans were only allowed to immigrate to the United States only after extensive screening. Participants were asked to rate their perceptions of the importance each type of screening (health, financial, criminal, and niceness) on a scale of 1 (*Strongly Disagree*) to 7 (*Strongly Agree*). For assurances, participants were asked to rate whether their degree of comfort with Krasneeans immigrating to the United States would be influenced if they knew that they would not have any contact with the Krasneeans, knew that the immigrating Krasneeans would adopt American norms and culture, or knew that the immigrating Krasneeans would come from an area without any viruses. Participants responded on a scale from 1 (*A lot less comfortable*) to 7 (*A lot more comfortable*).

Full results are reported starting on the next page. In terms of the importance of additional screening prior to immigration, pathogen disgust was associated with thinking that health, financial, and niceness screenings are important, but not that criminal screenings are important. Additionally, healthcare professionals thought that health and financial screenings were more important than did control participants. When pathogen disgust was predicted from all four screenings, controlling for participant group, health and niceness screenings were significantly associated with pathogen disgust. In terms of the effect assurances would have on participants’ comfort with Krasneean immigration, pathogen disgust was associated with more comfort with immigration if participants were assured that they would not come into contact with Krasneeans, that Krasneeans would adopt American norms, and that Krasneeans’ ecology is not pathogen rich. When pathogen disgust was predicted from all three assurances, controlling for participant group, the no contact assurance was the only assurance associated with pathogen disgust.

**Importance of Additional Screenings**

Table C

*Importance of Health Screenings*

| Dependent Variable | *b*(*SE*) | *t* | *p* | 95% CI | *r* |
| --- | --- | --- | --- | --- | --- |
| Primary Model |  |  |  |  |  |
| **Pathogen Disgust** | **0.34 (0.10)** | **3.37** | **.001** | **[0.14, 0.54]** | **.19** |
| **Career Group** | **0.67 (0.20)** | **3.34** | **.001** | **[0.28, 1.07]** | **.18** |
| Pathogen Disgust X Group | -0.35 (0.20) | -1.72 | .086 | [-0.74, 0.05] | -.01 |
| Ancillary Model |  |  |  |  |  |
| **Pathogen Disgust** | **0.31 (0.10)** | **3.07** | **.002** | **[0.11, 0.51]** | **.17** |
| **Career Group** | **0.59 (0.21)** | **2.86** | **.005** | **[0.18, 0.99]** | **.16** |
| Pathogen Disgust X Group | -0.29 (0.20) | -1.43 | .154 | [-0.69, 0.11] | -.08 |
| Age | 0.01 (0.01) | 0.56 | .576 | [-0.01, 0.02] | .03 |
| Gender | 0.04 (0.25) | 0.15 | .881 | [-0.45, 0.52] | .01 |
| **Political Orientation** | **0.13 (0.04)** | **3.02** | **.003** | **[0.04, 0.21]** | **.17** |
| Religiosity | -0.01 (0.04) | -0.22 | .826 | [-0.08, 0.07] | -.01 |

Table D

*Importance of Financial Screenings*

| Dependent Variable | *b*(*SE*) | *t* | *p* | 95% CI | *r* |
| --- | --- | --- | --- | --- | --- |
| Primary Model |  |  |  |  |  |
| **Pathogen Disgust** | **0.31 (0.10)** | **3.22** | **.001** | **[0.12, 0.49]** | **.18** |
| **Career Group** | **0.46 (0.19)** | **2.43** | **.016** | **[0.09, 0.84]** | **.13** |
| Pathogen Disgust X Group | 0.15 (0.19) | 0.77 | .444 | [-0.23, 0.52] | .04 |
| Ancillary Model |  |  |  |  |  |
| **Pathogen Disgust** | **0.25 (0.09)** | **2.79** | **.006** | **[0.07, 0.43]** | **.15** |
| Career Group | 0.32 (0.19) | 1.73 | .085 | [-0.05, 0.68] | .09 |
| Pathogen Disgust X Group | 0.23 (0.18) | 1.28 | .203 | [-0.13, 0.59] | .07 |
| Age | 0.00 (0.01) | 0.23 | .819 | [-0.01, 0.02] | .01 |
| Gender | 0.12 (0.22) | 0.52 | .604 | [-0.32, 0.55] | .03 |
| **Political Orientation** | **-0.03 (0.03)** | **0.37** | **<.001** | **[0.15, 0.30]** | **.31** |
| Religiosity | -0.03 (0.03) | -0.80 | .427 | [-0.09, 0.04] | -.04 |

Table E
*Importance of Criminal Screenings*

| Dependent Variable | *b*(*SE*) | *t* | *p* | 95% CI | *r* |
| --- | --- | --- | --- | --- | --- |
| Primary Model |  |  |  |  |  |
| Pathogen Disgust | 0.07 (0.07) | 0.88 | .378 | [-0.08, 0.21] | .05 |
| Career Group | 0.26 (0.15) | 1.78 | .076 | [-0.03, 0.55] | .10 |
| Pathogen Disgust X Group | -0.20 (0.15) | -1.33 | .186 | [-0.49, 0.10] | -.08 |
| Ancillary Model |  |  |  |  |  |
| Pathogen Disgust | 0.04 (0.07) | 0.53 | .596 | [-0.10, 0.18] | .03 |
| Career Group | 0.21 (0.15) | 1.45 | .149 | [-0.08, 0.51] | .08 |
| Pathogen Disgust X Group | -0.14(0.15) | -0.97 | .334 | [-0.43, 0.15] | -.05 |
| Age | 0.01 (0.01) | 1.96 | .051 | [0.00, 0.02] | .11 |
| Gender | -0.13 (0.18) | -0.72 | .472 | [-0.48, 0.22] | -.04 |
| **Political Orientation** | **0.11 (0.03)** | **3.76** | **<.001** | **[0.05, 0.17]** | **.21** |
| Religiosity | 0.00 (0.03) | -0.01 | .989 | [-0.05, 0.05] | -.00 |

Table F

*Importance of Niceness Screenings*

| Dependent Variable | *b*(*SE*) | *t* | *p* | 95% CI | *r* |
| --- | --- | --- | --- | --- | --- |
| Primary Model |  |  |  |  |  |
| **Pathogen Disgust** | **0.32 (0.10)** | **3.28** | **.001** | **[0.13, 0.51]** | **.18** |
| Career Group | 0.24 (0.20) | 1.25 | .213 | [-0.14, 0.63] | .07 |
| Pathogen Disgust X Group | -0.10 (0.20) | -0.52 | .601 | [-0.49, 0.28] | -.03 |
| Ancillary Model |  |  |  |  |  |
| **Pathogen Disgust** | **0.28 (0.10)** | **2.92** | **.004** | **[0.09, 0.47]** | **.16** |
| Career Group | 0.13 (0.20) | 0.63 | .529 | [-0.27, 0.51] | .03 |
| Pathogen Disgust X Group | -0.04 (0.19) | -0.19 | .847 | [-0.42, 0.34] | -.01 |
| Age | 0.01 (0.01) | 1.70 | .090 | [-0.00, 0.03] | .09 |
| Gender | 0.17 (0.24) | 0.49 | .625 | [-0.35, 0.58] | .03 |
| **Political Orientation** | **0.13 (0.04)** | **3.31** | **.001** | **[0.05, 0.21]** | **.18** |
| Religiosity | 0.01 (0.04) | 0.32 | .748 | [-0.06, 0.08] | .02 |

Table G

*Association between Pathogen Disgust and Importance of All Screenings*

| Dependent Variable | *b*(*SE*) | *t* | *p* | 95% CI | *r* |
| --- | --- | --- | --- | --- | --- |
| **Health Screening** | **0.09 (0.04)** | **2.26** | **.024** | **[0.01, 0.17]** | **.12** |
| Criminal Screening | -0.08 (0.06) | -1.53 | .128 | [-0.19, 0.02] | -.08 |
| Financial Screening | 0.08 (0.04) | 1.81 | .071 | [-0.01, 0.16] | .10 |
| **Niceness Screening** | **0.09 (0.04)** | **2.11** | **.035** | **[0.01, 0.17]** | **.12** |
| **Career Group** | **-0.43 (0.13)** | **-3.47** | **.001** | **[-0.68, -0.19]** | **-.19** |

**Effects of Assurances on Comfort**

Table H

Comfort if Assured No Contact with Krasneeans

| Dependent Variable | *b*(*SE*) | *t* | *p* | 95% CI | *r* |
| --- | --- | --- | --- | --- | --- |
| Primary Model |  |  |  |  |  |
| **Pathogen Disgust** | **0.32 (0.10)** | **3.28** | **.001** | **[0.13, 0.51]** | **.18** |
| Career Group | 0.24 (0.20) | 1.25 | .213 | [-0.14, 0.63] | .07 |
| Pathogen Disgust X Group | -0.10 (0.20) | -0.52 | .601 | [-0.49, 0.28] | -.03 |
| Ancillary Model |  |  |  |  |  |
| **Pathogen Disgust** | **0.33 (0.07)** | **4.51** | **<.001** | **[0.19, 0.47]** | **.25** |
| Career Group | 0.12 (0.15) | 0.82 | .413 | [-0.17, 0.42] | .05 |
| Pathogen Disgust X Group | -0.04 (0.15) | -0.30 | .763 | [-0.33, 0.24] | -0.02 |
| Age | 0.01 (0.01) | 1.91 | .057 | [0.00, 0.02] | .10 |
| Gender | -0.10 (0.18) | -0.53 | .597 | [-0.45, 0.26] | -.03 |
| Political Orientation | -0.00 (0.03) | -0.08 | .938 | [-0.06 0.06] | -.00 |
| Religiosity | 0.05 (0.03) | 1.81 | .071 | [-0.00, 0.10] | .10 |

Table I

*Comfort if Assured Krasneeans will Adopt Group Norms*

| Dependent Variable | *b*(*SE*) | *t* | *p* | 95% CI | *r* |
| --- | --- | --- | --- | --- | --- |
| Primary Model |  |  |  |  |  |
| **Pathogen Disgust** | **0.20 (0.09)** | **2.32** | **.021** | **[0.03, 0.36]** | **.13** |
| Career Group | 0.04 (0.17) | 0.22 | .827 | [-0.29, 0.37] | .01 |
| Pathogen Disgust X Group | -0.10 (0.17) | -0.60 | .547 | [-0.44, 0.23] | -.03 |
| Ancillary Model |  |  |  |  |  |
| Pathogen Disgust | 0.16 (0.08) | 1.92 | .056 | [-0.00, 0.32] | .11 |
| Career Group | -0.06 (0.17) | -0.35 | .729 | [-0.39, 0.27] | -.02 |
| Pathogen Disgust X Group | -0.03 (0.16) | -0.18 | .858 | [-0.35, 0.29] | -.01 |
| **Age** | **0.02 (0.01)** | **3.61** | **<.001** | **[0.01, 0.04]** | **.20** |
| Gender | 0.15 (0.20) | 0.77 | .443 | [-0.24, 0.55] | .04 |
| **Political Orientation** | **0.08 (0.03)** | **2.22** | **.027** | **[0.01, 0.14]** | **.12** |
| Religiosity | 0.03 (0.03) | 1.01 | .313 | [-0.03, 0.09] | .06 |

Table J

Comfort if Assured Krasneeans Come from a Non-Pathogen Rich Ecology

| Dependent Variable | *b*(*SE*) | *t* | *p* | 95% CI | *r* |
| --- | --- | --- | --- | --- | --- |
| Primary Model |  |  |  |  |  |
| **Pathogen Disgust** | **0.17 (0.08)** | **2.11** | **.036** | **[0.01, 0.33]** | **.12** |
| Career Group | -0.02 (0.16) | -0.15 | .885 | [-0.34, 0.30] | -.01 |
| Pathogen Disgust X Group | -0.09 (0.16) | -0.56 | .576 | [-0.41, 0.23] | -.03 |
| Ancillary Model |  |  |  |  |  |
| **Pathogen Disgust** | **0.17 (0.08)** | **2.02** | **.044** | **[0.00, 0.33]** | **.12** |
| Career Group | -0.08 (0.17) | -0.49 | .624 | [-0.42, 0.25] | -.03 |
| Pathogen Disgust X Group | -0.09 (0.17) | -0.53 | .598 | [-0.42, 0.24] | -.03 |
| Age | 0.00 (0.01) | 0.05 | .960 | [-0.01, 0.01] | .00 |
| Gender | 0.33 (0.20) | 1.60 | .111 | [-0.08, 0.73] | .09 |
| Political Orientation | -0.02 (0.04) | -0.42 | .673 | [-0.08, 0.05] | -.02 |
| Religiosity | 0.01 (0.03) | 0.29 | .771 | [-0.05, 0.07] | .02 |

Table K

*Association between Pathogen Disgust and Comfort following All Assurances*

| Dependent Variable | *b*(*SE*) | *t* | *p* | 95% CI | *r* |
| --- | --- | --- | --- | --- | --- |
| **No Contact** | **0.22 (0.05)** | **4.19** | **<.001** | **[0.12, 0.32]** | **.23** |
| Adopt Norms | -0.01 (0.05) | -0.19 | .848 | [-0.10, 0.09] | -.01 |
| Non-Pathogen Ecology | 0.07 (0.05) | 1.63 | .105 | [-0.02, 0.16] | .09 |
| **Career Group** | **-0.37 (0.12)** | **-2.99** | **.003** | **[-0.61, -0.13]** | **-.16** |

# **Exploratory analyses examining associations between sexual disgust and Krasneean immigration**

Table L

*Likeable*

| Dependent Variable | *b*(*SE*) | *t* | *p* | 95% CI | *r* |
| --- | --- | --- | --- | --- | --- |
| Primary Model |  |  |  |  |  |
| Career Group | 0.05(0.12) | 0.42 | .674 | [-0.19, 0.29] | .02 |
| Pathogen Disgust | -0.12(0.07) | -1.79 | .075 | [-0.24, 0.01] | -.10 |
| Control | -0.19(0.11) | -1.84 | .067 | [-0.40, 0.01] | -.10 |
| Healthcare Prof. | -0.04(0.08) | -0.50 | .616 | [-0.18, 0.11] | -.03 |
| Pathogen Disgust X Group | 0.16(0.13) | 1.21 | .228 | [-0.10, 0.41] | .07 |
| Sexual Disgust | -0.06(0.06) | -0.92 | .361 | [-0.18, 0.07] | -.05 |
| Control | 0.12(0.10) | 1.91 | .235 | [-0.08, 0.32] | .07 |
| **Healthcare Prof.** | **-0.24(0.08)** | **-3.00** | **.003** | **[-0.39, -0.08]** | **-.17** |
| **Sexual Disgust X Group** | **-0.63(0.13)** | **-2.79** | **.006** | **[-0.61, -0.10]** | **-.16** |
| Moral Disgust | 0.02(0.06) | 0.36 | .721 | [-0.10, 0.14] | .02 |
| Control | -0.04(0.09) | -0.43 | .665 | [-0.23, 0.15] | -.02 |
| Healthcare Prof. | 0.08(0.08) | 1.09 | .277 | [-0.07, 0.24] | .06 |
| Moral Disgust X Group | 0.13(0.12) | 1.03 | .305 | [-0.12, 0.37] | .06 |
| Ancillary Model |  |  |  |  |  |
| Career Group | 0.06(0.13) | 0.49 | .624 | [-0.19, 0.31] | .03 |
| Pathogen Disgust | -0.12(0.07) | -1.83 | .068 | [-0.25, 0.01] | -.10 |
| Control | -0.19(0.11) | -1.74 | .083 | [-0.40, 0.02] | -.10 |
| Healthcare Prof. | -0.06(0.08) | -0.74 | .462 | [-0.21, 0.09] | -.04 |
| Pathogen Disgust X Group | 0.13(0.06) | 1.00 | .316 | [-0.13, 0.39] | .06 |
| Sexual Disgust | -0.01(0.08) | -0.12 | .902 | [-0.16, 0.14] | -.01 |
| Control | 0.17(0.11) | 1.61 | .108 | [-0.04, 0.38] | .09 |
| **Healthcare Prof.** | **-0.36(0.13)** | **-2.84** | **.033** | **[-0.61, -0.11]** | **-.16** |
| **Sexual Disgust X Group** | **-0.36(0.13)** | **-2.84** | **.005** | **[-0.61, -0.11]** | **-.16** |
| Moral Disgust | 0.06(0.06) | 0.92 | .359 | [-0.07, 0.19] | .05 |
| Control | -0.00(0.10) | -0.03 | .973 | [-0.19, 0.19] | -.00 |
| Healthcare Prof. | 0.12(0.08) | 1.51 | .133 | [-0.04, 0.28] | .08 |
| Moral Disgust X Group | 0.13(0.12) | 1.02 | .309 | [-0.12, 0.37] | .06 |
| Age | -0.01(0.01) | -1.74 | .083 | [-0.02, 0.00] | -.01 |
| Gender | -0.12(0.16) | -0.74 | .461 | [-0.42, 0.19] | -.04 |
| Political Orientation | -0.05(0.03) | -1.92 | .056 | [-0.10, 0.00] | -.11 |
| Religiosity | 0.01(0.02) | 0.21 | .832 | [-0.04, 0.05] | .01 |

Table M

*Hostile*

| Dependent Variable | *b*(*SE*) | *t* | *p* | 95% CI | *r* |
| --- | --- | --- | --- | --- | --- |
| Primary Model |  |  |  |  |  |
| Career Group | 0.11(0.15) | 0.71 | .481 | [-0.19, 0.40] | .04 |
| **Pathogen Disgust** | **0.23(0.08)** | **2.99** | **.003** | **[0.08, 0.39]** | **.17** |
| **Control** | **0.41(0.13)** | **3.19** | **.002** | **[0.16, 0.66]** | **.18** |
| Healthcare Prof. | 0.06(0.09) | 0.68 | .499 | [-0.12, 0.24] | .04 |
| **Pathogen Disgust X Group** | **-0.34(0.16)** | **-2.20** | **.029** | **[-0.65, -0.04]** | **-.12** |
| Sexual Disgust | 0.12(0.08) | 1.55 | .121 | [-0.03, 0.27] | .09 |
| Control | 0.10(0.12) | 0.86 | .392 | [-0.14, 0.34] | .05 |
| Healthcare Prof. | 0.14(0.10) | 1.42 | .158 | [-0.05, 0.33] | .08 |
| Sexual Disgust X Group | 0.03(0.16) | 0.21 | .833 | [-0.27, 0.34] | .01 |
| Moral Disgust | -0.01(0.08) | -0.07 | .948 | [-0.15, 0.14] | -.00 |
| Control | -0.04(0.12) | -0.35 | .724 | [-0.27, 0.19] | -.02 |
| Healthcare Prof. | 0.03(0.10) | 0.33 | .745 | [-0.16, 0.22] | .02 |
| Moral Disgust X Group | 0.07(0.15) | 0.48 | .632 | [-0.22, 0.37] | .03 |
| Ancillary Model |  |  |  |  |  |
| Career Group | 0.07(0.15) | 0.45 | .656 | [-0.24, 0.37] | .03 |
| **Pathogen Disgust** | **0.23(0.08)** | **2.90** | **.004** | **[0.07, 0.39]** | **.16** |
| **Control** | **0.39(0.13)** | **3.05** | **.003** | **[0.14, 0.65]** | **.17** |
| Healthcare Prof. | 0.07(0.09) | 0.76 | .446 | [-0.11, 0.25] | .04 |
| **Pathogen Disgust X Group** | **-0.32(0.16)** | **-2.05** | **.042** | **[-0.63, -0.01]** | **-.11** |
| Sexual Disgust | 0.07(0.09) | 0.80 | .422 | [-0.11, 0.25] | .05 |
| Control | 0.06(0.13) | 0.44 | .662 | [-0.20, 0.31] | .02 |
| Healthcare Prof. | 0.09(0.11) | 0.83 | .410 | [-0.12, 0.30] | .05 |
| Sexual Disgust X Group | 0.03(0.16) | 0.21 | .832 | [-0.27, 0.34] | .01 |
| Moral Disgust | -0.00(0.08) | -0.05 | .961 | [-0.16, 0.15] | -.00 |
| Control | -0.04(0.12) | -0.30 | .766 | [-0.27, 0.20] | -.02 |
| Healthcare Prof. | 0.03(0.10) | 0.28 | .781 | [-0.17, 0.22] | .02 |
| Moral Disgust X Group | 0.06(0.15) | 0.42 | .676 | [-0.23, 0.36] | .02 |
| Age | -.00(.01) | -0.41 | .685 | [-0.02, 0.01] | -.02 |
| Gender | 0.00(0.19) | 0.01 | .993 | [-0.37, 0.38] | .00 |
| **Political Orientation** | **0.09(0.03)** | **2.79** | **.006** | **[0.03, 0.15]** | **.16** |
| Religiosity | -0.02(0.03) | -0.71 | .476 | [-0.08, 0.04] | -.04 |

Table N

*Trustworthy*

| Dependent Variable | *b*(*SE*) | *t* | *p* | 95% CI | *r* |
| --- | --- | --- | --- | --- | --- |
| Primary Model |  |  |  |  |  |
| Career Group | -0.01(0.12) | -0.07 | .946 | [-0.25, 0.23] | .00 |
| Pathogen Disgust | 0.00(0.06) | -0.02 | .986 | [-0.13, 0.12] | .00 |
| Pathogen Disgust X Group | 0.08(0.13) | 0.64 | .522 | [-0.17, 0.33] | .04 |
| Sexual Disgust | -0.07(0.06) | -1.15 | .251 | [-0.20, 0.05] | -.07 |
| Sexual Disgust X Group | -0.09(0.13) | -0.68 | .495 | [-0.34, 0.16] | -.04 |
| Moral Disgust | 0.00(0.06) | -0.05 | .959 | [-0.12, 0.12] | .00 |
| Moral Disgust X Group | 0.05(0.12) | 0.39 | .698 | [-0.19, 0.29] | .02 |
| Ancillary Model |  |  |  |  |  |
| Career Group | -0.04(0.12) | -0.29 | .769 | [-0.28, 0.21] | -.02 |
| Pathogen Disgust | 0.00(0.06) | -.01 | .995 | [-0.13, 0.13] | .00 |
| Pathogen Disgust X Group | 0.04(0.13) | 0.35 | .726 | [-0.20, 0.29] | .02 |
| Sexual Disgust | -0.04(0.07) | -0.56 | .580 | [-0.18, 0.10] | -.03 |
| Sexual Disgust X Group | -0.10(0.12) | -0.79 | .428 | [-0.34, 0.15] | -.05 |
| Moral Disgust | 0.02(0.06) | 0.34 | .732 | [-0.10, 0.15] | .02 |
| Moral Disgust X Group | 0.07(0.12) | 0.63 | .532 | [-0.16, 0.31] | .04 |
| Age | 0.00(0.01) | -0.85 | .395 | [-0.01, 0.01] | -.05 |
| Gender | 0.10(0.15) | 0.65 | .515 | [-0.20, 0.40] | .04 |
| **Political Orientation** | **-0.09(0.03)** | **-3.50** | **.001** | **[-0.14, -0.04]** | **-.20** |
| Religiosity | 0.03(0.02) | 1.29 | .199 | [-0.02, 0.08] | .07 |

Table O

*Open-Minded*

| Dependent Variable | *b*(*SE*) | *t* | *p* | 95% CI | *r* |
| --- | --- | --- | --- | --- | --- |
| Primary Model |  |  |  |  |  |
| Career Group | -0.03(0.17) | -0.15 | .882 | [-0.36, 0.31] | -.01 |
| Pathogen Disgust | 0.06(0.09) | 0.66 | .513 | [-0.12, 0.23] | .04 |
| Pathogen Disgust X Group | 0.00(0.18) | -0.01 | .989 | [-0.35, 0.34] | .00 |
| Sexual Disgust | -0.08(0.09) | -0.96 | .336 | [-0.26, 0.09] | -.06 |
| Sexual Disgust X Group | -0.05(0.17) | -0.27 | .788 | [-0.40, 0.30] | -.02 |
| Moral Disgust | -0.01(0.08) | -0.10 | .917 | [-0.17, 0.16] | -.01 |
| Moral Disgust X Group | -0.09(0.17) | -0.52 | .602 | [-0.42, 0.24] | -.03 |
| Ancillary Model |  |  |  |  |  |
| Career Group | -0.03(0.17) | -0.17 | .869 | [-0.37, 0.31] | -.01 |
| Pathogen Disgust | 0.02(0.09) | 0.18 | .854 | [-0.16, 0.19] | .01 |
| Pathogen Disgust X Group | -0.01(0.17) | -0.06 | .949 | [-0.35, 0.33] | .00 |
| Sexual Disgust | 0.04(0.10) | 0.36 | .717 | [-0.16, 0.24] | .02 |
| Sexual Disgust X Group | -0.05(0.17) | -0.29 | .769 | [-0.39, 0.29] | -.02 |
| Moral Disgust | 0.05(0.09) | 0.55 | .585 | [-0.12, 0.22] | .03 |
| Moral Disgust X Group | -0.06(0.17) | -0.37 | .714 | [-0.39, 0.27] | -.02 |
| Age | -0.01(0.01) | -0.88 | .380 | [-0.02, 0.01] | -.05 |
| **Gender** | **0.16(0.21)** | **0.75** | **.015** | **[-0.26, 0.57]** | **.04** |
| Political Orientation | -0.09(0.04) | -2.44 | .185 | [-0.15, -0.02] | -.14 |
| Religiosity | -0.04(0.03) | -1.33 | .457 | [-0.11, 0.02] | -.08 |

Table P

*Ignorant*

| Dependent Variable | *b*(*SE*) | *t* | *p* | 95% CI | *r* |
| --- | --- | --- | --- | --- | --- |
| Primary Model |  |  |  |  |  |
| Career Group | -0.03(0.16) | -0.20 | .842 | [-0.34, 0.28] | -.01 |
| Pathogen Disgust | 0.16(0.08) | 1.93 | .055 | [0.00, 0.31] | .11 |
| Pathogen Disgust X Group | -0.14(0.16) | -0.84 | .399 | [-0.45, 0.18] | -.05 |
| Sexual Disgust | -0.02(0.08) | -0.26 | .793 | [-0.18, 0.14] | -.02 |
| Sexual Disgust X Group | 0.08(0.16) | 0.49 | .625 | [-0.24, 0.39] | .03 |
| Moral Disgust | 0.02(0.08) | 0.28 | .779 | [-0.13, 0.17] | .02 |
| Moral Disgust X Group | 0.20(0.15) | 1.31 | .192 | [-0.10, 0.50] | .07 |
| Ancillary Model |  |  |  |  |  |
| Career Group | 0.01(0.15) | 0.06 | .956 | [-0.30, 0.31] | .00 |
| Pathogen Disgust | 0.14(0.08) | 1.77 | .078 | [-0.02, 0.30] | .10 |
| Pathogen Disgust X Group | -0.13(0.16) | -0.82 | .413 | [-0.44, 0.18] | -.05 |
| Sexual Disgust | 0.05(0.10) | 0.59 | .558 | [-0.13, 0.23] | .03 |
| Sexual Disgust X Group | 0.09(0.16) | 0.56 | .574 | [-0.22, 0.39] | .03 |
| Moral Disgust | -0.01(0.08) | -0.07 | .947 | [-0.16, 0.15] | .00 |
| Moral Disgust X Group | 0.16(0.15) | 1.04 | .299 | [-0.14, 0.45] | .06 |
| Age | 0.00(0.01) | 0.52 | .603 | [-0.01, 0.02] | .03 |
| **Gender** | **-0.64(0.19)** | **-3.38** | **.001** | **[-1.02, -0.27]** | **-.19** |
| Political Orientation | 0.06(0.03) | 1.95 | .052 | [0.00, 0.12] | .11 |
| Religiosity | -0.03(0.03) | -0.98 | .326 | [-0.09, 0.03] | -.06 |

Table Q

*Poor*

| Dependent Variable | *b*(*SE*) | *t* | *p* | 95% CI | *r* |
| --- | --- | --- | --- | --- | --- |
| Primary Model |  |  |  |  |  |
| Career Group | -0.05(0.18) | -0.30 | .766 | [-0.41, 0.30] | -.02 |
| Pathogen Disgust | 0.02(0.09) | 0.22 | .829 | [-0.17, 0.21] | .01 |
| Pathogen Disgust X Group | 0.03(0.19) | 0.16 | .874 | [-0.34, 0.40] | .01 |
| Sexual Disgust | 0.03(0.09) | 0.31 | .754 | [-0.15, 0.21] | .02 |
| Sexual Disgust X Group | -0.20(0.19) | -1.05 | .294 | [-0.56, 0.17] | -.06 |
| Moral Disgust | 0.07(0.09) | 0.79 | .433 | [-0.11, 0.25] | .05 |
| Moral Disgust X Group | 0.09(0.18) | 0.49 | .621 | [-0.26, 0.44] | .03 |
| Ancillary Model |  |  |  |  |  |
| Career Group | 0.01(0.18) | 0.07 | .948 | [-0.35, 0.37] | .00 |
| Pathogen Disgust | 0.03(0.10) | 0.31 | .755 | [-0.16, 0.22] | .02 |
| Pathogen Disgust X Group | 0.05(0.19) | 0.25 | .806 | [-0.32, 0.41] | .01 |
| Sexual Disgust | 0.07(0.11) | 0.68 | .496 | [-0.14, 0.29] | .04 |
| Sexual Disgust X Group | -0.19(0.18) | -1.06 | .291 | [-0.56, 0.17] | -.06 |
| Moral Disgust | 0.02(0.09) | 0.24 | .810 | [-0.16, 0.21] | .01 |
| Moral Disgust X Group | 0.03(0.18) | 0.17 | .864 | [-0.32, 0.38] | .01 |
| Age | 0.00(0.01) | 0.48 | .632 | [-0.01, 0.02] | .03 |
| Gender | -0.76(0.23) | -3.36 | .274 | [-1.20, -0.31] | -.19 |
| Political Orientation | 0.04(0.04) | 1.10 | .955 | [-0.03, 0.11] | .06 |
| **Religiosity** | **0.00(0.04)** | **0.06** | **.001** | **[-0.07, 0.07]** | **.00** |

Table R

*Unclean*

| Dependent Variable | *b*(*SE*) | *t* | *p* | 95% CI | *r* |
| --- | --- | --- | --- | --- | --- |
| Primary Model |  |  |  |  |  |
| Career Group | -0.07(0.17) | -0.43 | .669 | [-0.41, 0.27] | -.02 |
| **Pathogen Disgust** | **0.26(0.09)** | **2.94** | **.003** | **[0.09, 0.44]** | **.17** |
| Pathogen Disgust X Group | 0.00(0.18) | -0.02 | .984 | [-0.36, 0.35] | .00 |
| Sexual Disgust | 0.06(0.09) | 0.71 | .481 | [-0.11, 0.24] | .04 |
| Sexual Disgust X Group | -0.15(0.18) | -0.87 | .387 | [-0.50, 0.20] | -.05 |
| Moral Disgust | -0.04(0.09) | -0.46 | .643 | [-0.21, 0.13] | -.03 |
| Moral Disgust X Group | 0.05(0.17) | 0.29 | .772 | [-0.29, 0.39] | .02 |
| Ancillary Model |  |  |  |  |  |
| Career Group | -0.04(0.18) | -0.23 | .816 | [-0.39, 0.31] | -.01 |
| **Pathogen Disgust** | **0.30(0.09)** | **3.24** | **.001** | **[0.12, 0.48]** | **.18** |
| Pathogen Disgust X Group | 0.00(0.18) | -0.02 | .985 | [-0.36, 0.35] | .00 |
| Sexual Disgust | -0.01(0.10) | -0.05 | .958 | [-0.21, 0.20] | .00 |
| Sexual Disgust X Group | -0.15(0.18) | -0.86 | .391 | [-0.50, 0.20] | -.05 |
| Moral Disgust | -0.06(0.09) | -0.70 | .486 | [-0.24, 0.11] | -.04 |
| Moral Disgust X Group | 0.00(0.17) | 0.02 | .987 | [-0.33, 0.34] | .00 |
| Age | 0.00(0.01) | -0.24 | .813 | [-0.02, 0.01] | -.01 |
| Gender | -0.36(0.22) | -1.64 | .101 | [-0.79, 0.07] | -.09 |
| Political Orientation | 0.06(0.04) | 1.59 | .113 | [-0.01, 0.13] | .09 |
| Religiosity | 0.03(0.03) | 1.00 | .318 | [-0.03, 0.10] | .06 |

# **Exploratory analyses examining associations between sexual disgust and endorsement of moral foundations**

Table S

*Endorsement of Binding Moral Foundations*

| Dependent Variable | *b*(*SE*) | *t* | *p* | 95% CI | *r* |
| --- | --- | --- | --- | --- | --- |
| Primary Model |  |  |  |  |  |
| Individualizing | 0.04(0.07) | 0.50 | .619 | [-0.10, 0.17] | .03 |
| Career Group | 0.14(0.11) | 1.26 | .210 | [-0.08, 0.37] | .02 |
| Pathogen Disgust | 0.12(0.06) | 1.92 | .056 | [-0.003, 0.23] | .09 |
| Pathogen Disgust X Group | -0.04(0.12) | -0.32 | .750 | [-0.27, 0.20] | -.02 |
| **Sexual Disgust** | **0.38(0.06)** | **6.32** | **<.001** | **[0.26, 0.49]** | **.31** |
| Sexual Disgust X Group | -0.18(0.12) | -1.56 | .121 | [-0.41, 0.05] | -.08 |
| **Moral Disgust** | **0.23(0.06)** | **4.05** | **<.001** | **[0.12, 0.35]** | **0.20** |
| Control | 0.11(0.09) | 1.25 | .214 | [-0.06, 0.28] | .06 |
| Healthcare Prof. | **0.36(0.07)** | **4.91** | **<.001** | **[0.21, 0.50]** | **.24** |
| **Moral Disgust X Group** | **0.25(0.11)** | **2.20** | **.028** | **[0.03, 0.47]** | **.11** |
| Ancillary Model |  |  |  |  |  |
| **Individualizing** | **0.36(0.06)** | **6.23** | **<.001** | **[0.25, 0.48]** | **.23** |
| Career Group | 0.19(0.09) | 2.08 | .039 | [0.01, 0.36] | .08 |
| **Pathogen Disgust** | **0.15(0.05)** | **3.24** | **.001** | **[0.06, 0.24]** | **.12** |
| Pathogen Disgust X Group | -0.01(0.09) | -0.07 | .944 | [-0.19, 0.17] | -.003 |
| **Sexual Disgust** | **0.14(0.05)** | **2.62** | **.009** | **[0.04. 0.24]** | **.10** |
| **Control** | **0.23(0.08)** | **3.02** | **.003** | **[0.08, 0.38]** | **.11** |
| Healthcare Prof. | 0.05(0.06) | 0.80 | .427 | [-0.07, 0.17] | .03 |
| **Sexual Disgust X Group** | **-0.18(0.09)** | **-1.97** | **.049** | **[-0.35, -0.0004]** | **-.07** |
| Moral Disgust | 0.09(0.05) | 1.91 | .057 | [-0.003, 0.18] | .07 |
| Moral Disgust X Group | 0.15(0.09) | 1.75 | .080 | [-0.02, 0.32] | .07 |
| **Age** | **0.01(0.004)** | **2.14** | **.033** | **[0.001, 0.02]** | **.08** |
| **Gender** | **-0.30(0.11)** | **-2.66** | **.008** | **[-0.52, -0.08]** | **-.10** |
| **Political Orientation** | **0.20(0.02)** | **10.46** | **<.001** | **[0.16, 0.23]** | **.39** |
| **Religiosity** | **0.08(0.02)** | **4.96** | **<.001** | **[0.05, 0.12]** | **.18** |

Table T

*Endorsement of Individualizing Moral Foundations*

| Dependent Variable | *b*(*SE*) | *t* | *p* | 95% CI | *r* |
| --- | --- | --- | --- | --- | --- |
| Primary Model |  |  |  |  |  |
| Binding | 0.02(0.05) | 0.50 | .619 | [-0.07, 0.11] | .03 |
| Career Group | -0.06(0.09) | -0.64 | .521 | [-0.24, 0.12] | -.04 |
| **Pathogen Disgust** | **0.15(0.05)** | **3.19** | **.002** | **[0.06, 0.25]** | **.17** |
| Pathogen Disgust X Group | 0.01(0.10) | 0.14 | .890 | [-0.17, 0.20] | .01 |
| **Sexual Disgust** | **-0.14(0.05)** | **-2.82** | **.005** | **[-0.24, -0.04]** | **-.15** |
| Sexual Disgust X Group | 0.02(0.10) | 0.16 | .871 | [-0.17, 0.20] | .01 |
| **Moral Disgust** | **0.17(0.05)** | **3.59** | **<.001** | **[0.08, 0.26]** | **.19** |
| Moral Disgust X Group | 0.01(0.09) | 0.10 | .921 | [-0.17, 0.19] | .01 |
| Ancillary Model |  |  |  |  |  |
| **Binding** | **0.32(0.05)** | **6.23** | **<.001** | **[0.22, 0.42]** | **.30** |
| Career Group | -0.13(0.08) | -1.50 | .134 | [-0.29, 0.04] | -.07 |
| Pathogen Disgust | 0.07(0.04) | 1.48 | .141 | [-0.02, 0.15] | .07 |
| Pathogen Disgust X Group | -0.01(0.09) | -0.07 | .948 | [-0.17, 0.16] | -.003 |
| Sexual Disgust | -0.08(0.05) | -1.66 | .098 | [-0.18, 0.02] | -.08 |
| Sexual Disgust X Group | 0.06(0.08) | 0.74 | .458 | [-0.10, 0.23] | .04 |
| **Moral Disgust** | **0.16(0.04)** | **3.61** | **<.001** | **[0.07, 0.24]** | **.17** |
| Moral Disgust X Group | 0.01(0.08) | 0.07 | .941 | [-0.15, 0.17] | .004 |
| Age | -0.01(0.003) | -1.58 | .115 | [-0.01, 0.001] | -.08 |
| **Gender** | **0.29(0.10)** | **2.81** | **.005** | **[0.09, 0.49]** | **.13** |
| **Political Orientation** | **-0.14(0.02)** | **-7.68** | **<.001** | **[-0.18, -0.11]** | **-.37** |
| **Religiosity** | **-0.04(0.02)** | **-2.64** | **.009** | **[-0.08, -0.01]** | **-.13** |

# **Mediation models to examine whether disgust sensitivity mediates the group differences in political orientation and religiosity**

We used PROCESS (Model 4) to examine whether the between group difference in political orientation (i.e., healthcare professionals reporting being more conservative than control participants) was mediated by disgust sensitivity. In this model, we included pathogen, sexual, and moral disgust as possible mediators. Statistics for Path A and Path B are reported in Figure S1. The indirect effect via sexual disgust was significant, *b* = 0.29, *SE* = 0.13, 95% CI [0.06, 0.57]; neither the indirect effect via pathogen disgust, *b* = 0.07, *SE* = 0.05, 95% CI [-0.02, 0.21], nor moral disgust, *b* = 0.04, *SE* = 0.05, 95% CI [-0.04, 0.15], were significant.

We next estimated the same model for religiosity (see Figure S2). In addition to a significant indirect effect via sexual disgust, *b* = 0.44, *SE* = 0.19, 95% CI [0.08, 0.82], there was a significant indirect effect via pathogen disgust, *b* = 0.19, *SE* = 0.08, 95% CI [0.04, 0.37]. Again, the indirect effect via moral disgust was not significant, *b* = 0.06, *SE* = 0.05, 95% CI [-0.03, 0.18].


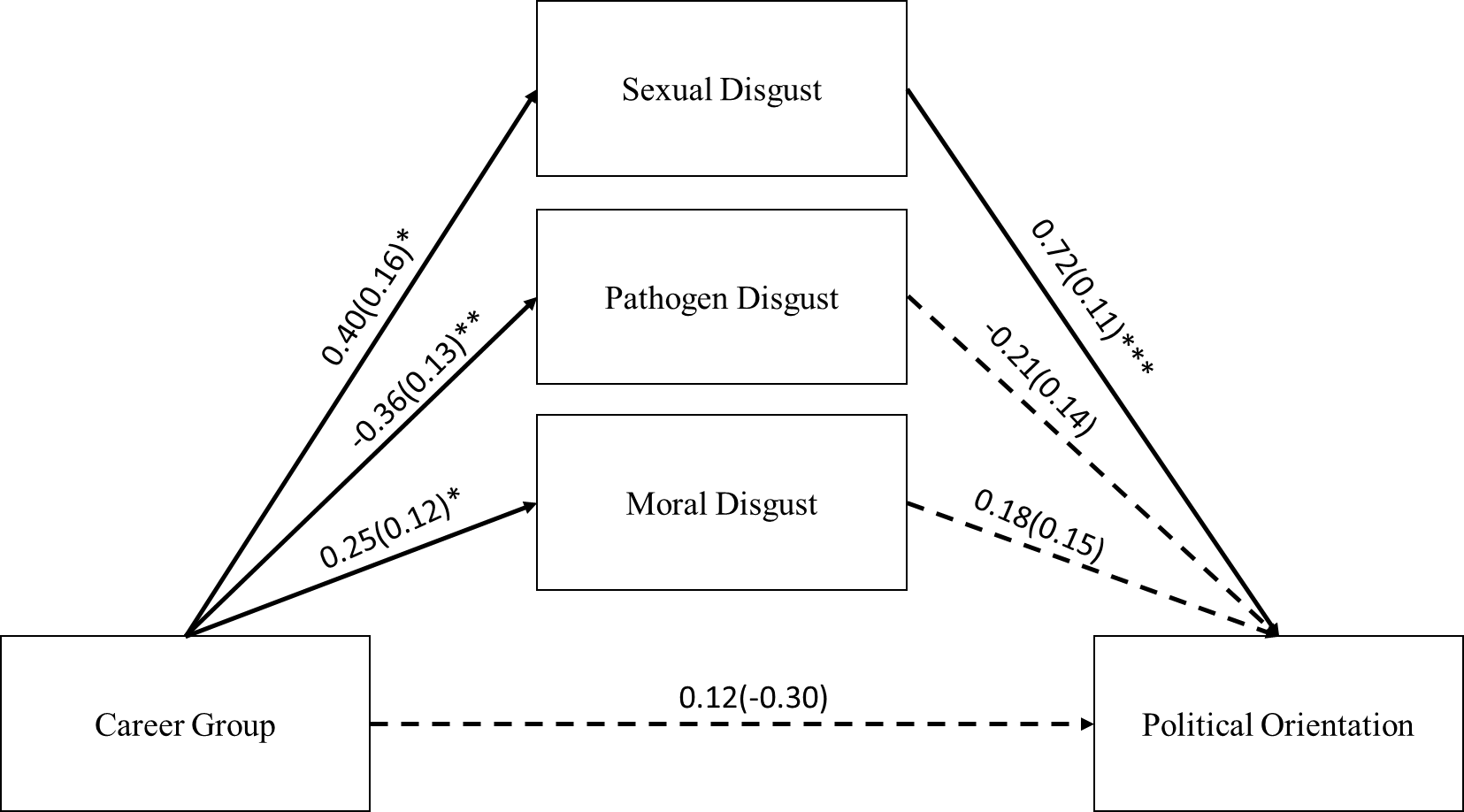


**Figure S1.** The statistics presented are the unstandardized coefficients and standard errors (in parentheses). *** *p* < .001, ** *p* < .01, * *p* < .05


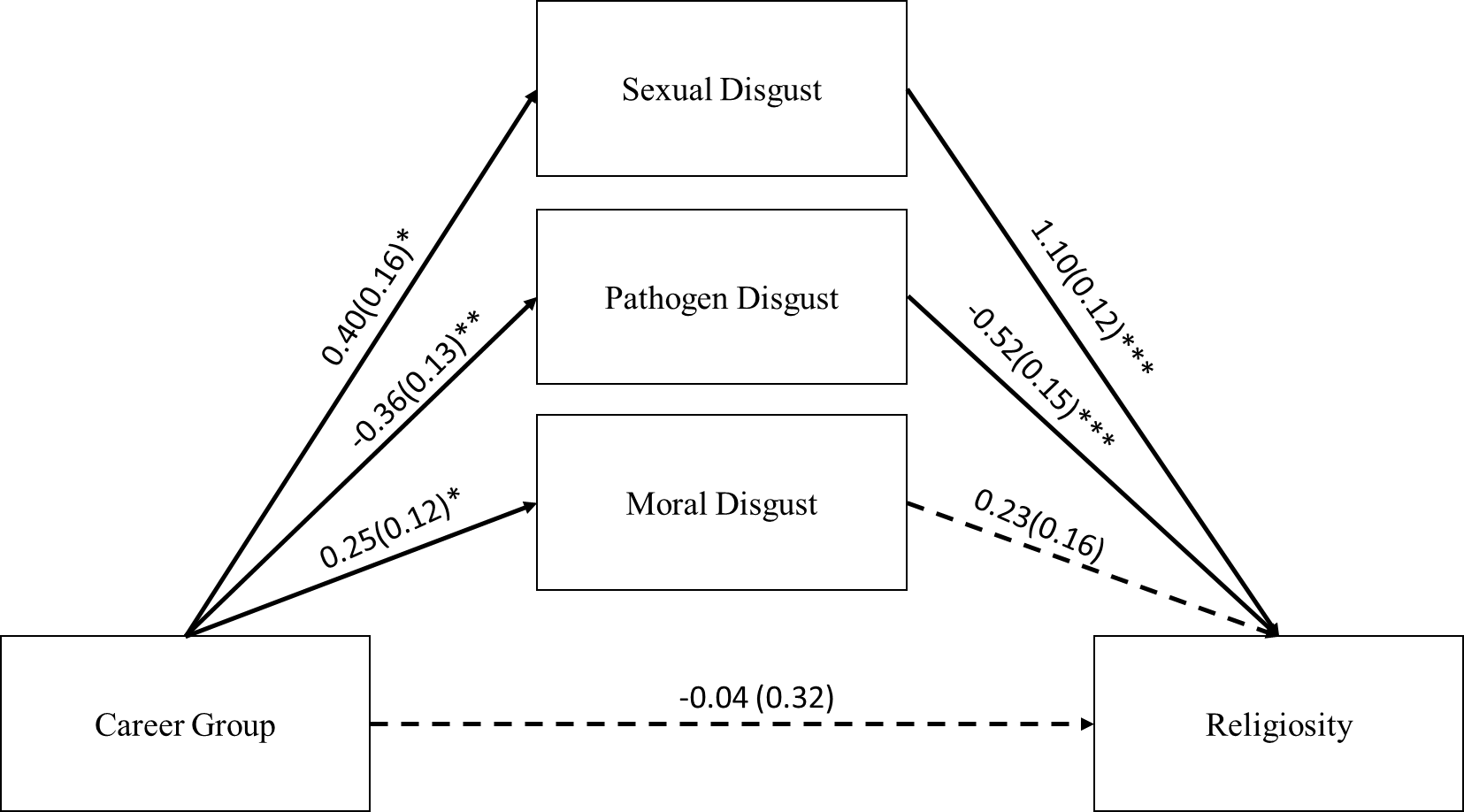


**Figure S2.** The statistics presented are the unstandardized coefficients and standard errors (in parentheses). *** *p* < .001, ** *p* < .01, * *p* < .05

# **Assessment of categorization bias for elderly targets**

*Social Categorization Bias.* To assess social avoidance biases against elderly targets, we first created minimal groups using procedures adapted from prior research (Miller et al., 2010; Makhanova et al., 2015). Participants completed a personality test and were randomly assigned to either green or orange personality type. Participants were informed that there were two different personality types but were not of any characteristics that made them different. Next, participants were shown 20 target photographs and were instructed to use their intuition to classify the individuals as either green or orange personality types. In random order, all participants saw 20 photographs of men that had neutral facial expressions and were either young or old and either Black or White (Makhanova et al., 2015). As in recent research (Makhanova et al., in press), we conducted mixed effects analyses to examine effects of target age (-0.5 = young, 0.5 = elderly), target race (-0.5 = white, 0.5 = black), career in medicine (or not), and pathogen disgust—and all interactions—on target outgroup categorization.

Model results are presented starting on the next page. Overall, there were marginal interactions between effect of both target race and target age and pathogen disgust that additionally depended on career group. Among control participants, both effects of target race (marginal) and target age (significant) depended on participants’ pathogen disgust. Among healthcare professionals, those interactions were not significant and there was only a main effect of pathogen disgust; healthcare professionals with higher pathogen disgust were more likely to categorize everyone as outgroup members. Notably, these associations were not robust in ancillary analyses that excluded participants who (1) did not self-identify as White and (2) were over 50 years old. The 3-way interaction with target age was no longer significant. Among control participants, there was a marginal interaction between target race and pathogen disgust. Among healthcare professionals, there was a marginal main effect of pathogen disgust.

Because of these inconsistencies, any effects should be interpreted with caution and replicated before any conclusions can be drawn about effects of pathogen disgust and career in medicine on bias in social categorization.

**References**

Makhanova, A., Eubanks, A. D., & Maner, J. K. (in press). Does pathogen avoidance affect intergroup categorization? Replication of Makhanova, Miller, and Maner (2015). *Evolutionary Behavioral Sciences*. Advanced online publication.

Makhanova, A., Miller, S. L., & Maner, J. K. (2015). Germs and the out-group: Chronic and situational disease concerns affect intergroup categorization. *Evolutionary Behavioral Sciences*, *9*(1), 8-19.

Miller, S. L., Maner, J. K., & Becker, D. V. (2010). Self-protective biases in group categorization: Threat cues shape the psychological boundary between “us” and “them”. *Journal of Personality and Social Psychology*, *99*(1), 62-77.

**All Participants**

Data and script for these analyses will be shared on reasonable request to the corresponding author.

**4-way interaction:**

Estimate Std. Error z value Pr(>|z|)

(Intercept) 0.172139 0.035634 4.831 1.36e-06 ***

target_age 0.082539 0.066967 1.233 0.2177

target_race -0.408029 0.063338 -6.442 1.18e-10 ***

mid_medwhole 0.026821 0.071268 0.376 0.7067

Zpathdisg2 0.002249 0.035737 0.063 0.9498

target_age:target_race 0.213960 0.112096 1.909 0.0563 .

target_age:Zpathdisg2 0.103023 0.067181 1.534 0.1251

target_race:Zpathdisg2 0.085586 0.063556 1.347 0.1781

target_age:mid_medwhole -0.068821 0.133934 -0.514 0.6074

target_race:mid_medwhole 0.110577 0.126677 0.873 0.3827

mid_medwhole:Zpathdisg2 0.183920 0.071474 2.573 0.0101 *

target_age:target_race:Zpathdisg2 -0.031641 0.112568 -0.281 0.7786

target_age:target_race:mid_medwhole 0.126484 0.224192 0.564 0.5726

target_age:mid_medwhole:Zpathdisg2 -0.256185 0.134361 -1.907 0.0566 .

target_race:mid_medwhole:Zpathdisg2 -0.230427 0.127113 -1.813 0.0699 .

target_age:target_race:mid_medwhole:Zpathdisg2 0.152570 0.225137 0.678 0.4980

**All 3-way interactions:**

Estimate Std. Error z value Pr(>|z|)

(Intercept) 0.171999 0.035632 4.827 1.38e-06 ***

target_age 0.083136 0.066967 1.241 0.21444

target_race -0.407832 0.063350 -6.438 1.21e-10 ***

mid_medwhole 0.027023 0.071264 0.379 0.70455

Zpathdisg2 0.002287 0.035735 0.064 0.94897

target_age:target_race 0.199199 0.109943 1.812 0.07001 .

target_age:Zpathdisg2 0.102611 0.067183 1.527 0.12668

target_race:Zpathdisg2 0.085460 0.063568 1.344 0.17882

target_age:mid_medwhole -0.069652 0.133942 -0.520 0.60305

target_race:mid_medwhole 0.110037 0.126699 0.868 0.38512

mid_medwhole:Zpathdisg2 0.184252 0.071471 2.578 0.00994 **

target_age:target_race:Zpathdisg2 -0.006978 0.106499 -0.066 0.94776

target_age:target_race:mid_medwhole 0.143572 0.222734 0.645 0.51919

target_age:mid_medwhole:Zpathdisg2 -0.255607 0.134359 -1.902 0.05712 .

target_race:mid_medwhole:Zpathdisg2 -0.231185 0.127137 -1.818 0.06900 .

**Drop the NS 3-way interactions:**

Estimate Std. Error z value Pr(>|z|)

(Intercept) 0.17184 0.03563 4.823 1.41e-06 ***

target_age 0.08300 0.06696 1.239 0.21519

target_race -0.40761 0.06335 -6.434 1.24e-10 ***

mid_medwhole 0.02769 0.07126 0.389 0.69763

Zpathdisg2 0.00210 0.03573 0.059 0.95313

target_age:target_race 0.22298 0.10361 2.152 0.03139 *

target_age:Zpathdisg2 0.10311 0.06717 1.535 0.12479

target_race:Zpathdisg2 0.08576 0.06357 1.349 0.17732

target_age:mid_medwhole -0.06968 0.13392 -0.520 0.60288

target_race:mid_medwhole 0.10880 0.12671 0.859 0.39049

mid_medwhole:Zpathdisg2 0.18444 0.07147 2.581 0.00986 **

target_age:mid_medwhole:Zpathdisg2 -0.25624 0.13435 -1.907 0.05649 .

target_race:mid_medwhole:Zpathdisg2 -0.23166 0.12714 -1.822 0.06845 .

**Among Healthcare professionals:**

Estimate Std. Error z value Pr(>|z|)

(Intercept) 0.18568 0.04075 4.556 5.21e-06 ***

target_age 0.04816 0.07657 0.629 0.52941

target_race -0.35321 0.07241 -4.878 1.07e-06 ***

medicals 0.02768 0.07126 0.389 0.69764

Zpathdisg2 0.09432 0.04164 2.265 0.02350 *

target_age:target_race 0.22298 0.10361 2.152 0.03139 *

target_age:Zpathdisg2 -0.02501 0.07825 -0.320 0.74924

target_race:Zpathdisg2 -0.03007 0.07401 -0.406 0.68454

target_age:medicals -0.06968 0.13392 -0.520 0.60287

target_race:medicals 0.10880 0.12671 0.859 0.39050

medicals:Zpathdisg2 0.18444 0.07147 2.581 0.00986 **

target_age:medicals:Zpathdisg2 -0.25624 0.13435 -1.907 0.05649 .

target_race:medicals:Zpathdisg2 -0.23166 0.12714 -1.822 0.06845

**Among Control Participants:**

Estimate Std. Error z value Pr(>|z|)

(Intercept) 0.15800 0.05846 2.703 0.00687 **

target_age 0.11783 0.10987 1.072 0.28353

target_race -0.46201 0.10398 -4.443 8.86e-06 ***

medwhole 0.02769 0.07126 0.389 0.69763

Zpathdisg2 -0.09012 0.05808 -1.552 0.12077

target_age:target_race 0.22298 0.10361 2.152 0.03139 *

target_age:Zpathdisg2 0.23123 0.10920 2.117 0.03423 *

target_race:Zpathdisg2 0.20159 0.10338 1.950 0.05118 .

target_age:medwhole -0.06967 0.13392 -0.520 0.60289

target_race:medwhole 0.10881 0.12671 0.859 0.39049

medwhole:Zpathdisg2 0.18444 0.07147 2.581 0.00986 **

target_age:medwhole:Zpathdisg2 -0.25624 0.13435 -1.907 0.05649 .

target_race:medwhole:Zpathdisg2 -0.23165 0.12714 -1.822 0.06845 .

**White Participants under 50 Years Old**

**4-way Interaction:**

Estimate Std. Error z value Pr(>|z|)

(Intercept) 0.12001 0.03879 3.094 0.00198 **

target_age 0.07049 0.07870 0.896 0.37046

target_race -0.36690 0.07656 -4.793 1.65e-06 ***

mid_medwhole 0.10272 0.07758 1.324 0.18549

Zpathdisg2 0.00615 0.04001 0.154 0.87785

target_age:target_race 0.28334 0.13268 2.136 0.03271 *

target_age:Zpathdisg2 0.11848 0.08117 1.460 0.14441

target_race:Zpathdisg2 0.06007 0.07897 0.761 0.44686

target_age:mid_medwhole -0.05214 0.15741 -0.331 0.74048

target_race:mid_medwhole 0.08206 0.15311 0.536 0.59201

mid_medwhole:Zpathdisg2 0.13536 0.08002 1.692 0.09073 .

target_age:target_race:Zpathdisg2 -0.02290 0.13694 -0.167 0.86721

target_age:target_race:mid_medwhole 0.25444 0.26536 0.959 0.33764

target_age:mid_medwhole:Zpathdisg2 -0.13952 0.16235 -0.859 0.39015

target_race:mid_medwhole:Zpathdisg2 -0.30304 0.15793 -1.919 0.05501 .

target_age:target_race:mid_medwhole:Zpathdisg2 0.25226 0.27388 0.921 0.35702

**Drop the NS 3-way interactions:**

Estimate Std. Error z value Pr(>|z|)

(Intercept) 0.120381 0.038781 3.104 0.00191 **

target_age 0.083125 0.077659 1.070 0.28445

target_race -0.367981 0.076565 -4.806 1.54e-06 ***

mid_medwhole 0.103541 0.077578 1.335 0.18199

Zpathdisg2 0.004335 0.039956 0.108 0.91360

target_age:target_race 0.310345 0.121095 2.563 0.01038 *

target_age:Zpathdisg2 0.095448 0.076587 1.246 0.21267

target_race:Zpathdisg2 0.063539 0.078876 0.806 0.42050

target_age:mid_medwhole -0.062148 0.157392 -0.395 0.69294

target_race:mid_medwhole 0.080843 0.153162 0.528 0.59762

mid_medwhole:Zpathdisg2 0.146341 0.079201 1.848 0.06464 .

target_race:mid_medwhole:Zpathdisg2 -0.324441 0.156415 -2.074 0.03806 *

**Among Healthcare professionals:**

Estimate Std. Error z value Pr(>|z|)

(Intercept) 0.17215 0.04340 3.966 7.3e-05 ***

target_age 0.05205 0.08781 0.593 0.553344

target_race -0.32756 0.08568 -3.823 0.000132 ***

medicals 0.10354 0.07758 1.335 0.181984

Zpathdisg2 0.07751 0.04594 1.687 0.091601 .

target_age:target_race 0.31035 0.12109 2.563 0.010382 *

target_age:Zpathdisg2 0.09545 0.07659 1.246 0.212666

target_race:Zpathdisg2 -0.09868 0.09073 -1.088 0.276740

target_age:medicals -0.06215 0.15739 -0.395 0.692947

target_race:medicals 0.08085 0.15316 0.528 0.597599

medicals:Zpathdisg2 0.14634 0.07920 1.848 0.064641 .

target_race:medicals:Zpathdisg2 -0.32444 0.15641 -2.074 0.038058 *

**Control Participants:**

Estimate Std. Error z value Pr(>|z|)

(Intercept) 0.06861 0.06429 1.067 0.28590

target_age 0.11420 0.12937 0.883 0.37738

target_race -0.40841 0.12693 -3.217 0.00129 **

medwhole 0.10354 0.07758 1.335 0.18198

Zpathdisg2 -0.06883 0.06495 -1.060 0.28924

target_age:target_race 0.31034 0.12109 2.563 0.01038 *

target_age:Zpathdisg2 0.09545 0.07659 1.246 0.21266

target_race:Zpathdisg2 0.22576 0.12824 1.761 0.07832 .

target_age:medwhole -0.06215 0.15739 -0.395 0.69294

target_race:medwhole 0.08085 0.15316 0.528 0.59761

medwhole:Zpathdisg2 0.14634 0.07920 1.848 0.06464 .

target_race:medwhole:Zpathdisg2 -0.32445 0.15641 -2.074 0.03805 *
